# Contemporary definitions of infant growth failure and neurodevelopmental and behavioral outcomes in extremely premature infants at two years of age

**DOI:** 10.1038/s41372-023-01852-9

**Published:** 2024-01-09

**Authors:** Katie M. Strobel, Thomas R. Wood, Gregory C. Valentine, Kendell R. German, Semsa Gogcu, D. Taylor Hendrixson, Sarah E. Kolnik, Janessa B. Law, Dennis E. Mayock, Bryan A. Comstock, Patrick J. Heagerty, Sandra E. Juul

**Affiliations:** 1 Division of Neonatology, Department of Pediatrics, University of Washington, Seattle, WA USA; 2 Division of Neonatology, Department of Pediatrics, Wake Forest School of Medicine, Winston-Salem, NC USA; 3 Department of Biostatistics, University of Washington, Seattle, WA USA

**Keywords:** Outcomes research, Prognostic markers

## Abstract

**Background:**

Associations of 2-year neurodevelopmental and behavioral outcomes with growth trajectories of preterm infants are unknown.

**Methods:**

This secondary analysis of a preterm cohort examined in-hospital and discharge to 2-year changes in anthropometric z-scores. Two-year follow-up included Bayley Scales of Infant Development (BSID-III) and Child Behavior Checklist.

**Results:**

Among 590 infants, adjusted in-hospital growth was not associated with any BSID-III subscale. Occipitofrontal circumference (OFC) growth failure (GF) in-hospital was associated with increased adjusted odds of attention problems (aOR 1.65 [1.03, 2.65]), aggressive behavior (aOR 2.34 [1.12, 4.89]), and attention-deficit-hyperactivity symptoms (aOR 1.86 [1.05, 3.30]). Infants with OFC GF at 2 years had lower adjusted BSID-III language scores (−4.0 [−8.0, −0.1]), increased odds of attention problems (aOR 2.29 [1.11, 4.74]), aggressive behavior (aOR 3.09 [1.00, 9.56]), and externalizing problems (aOR 3.01 [1.07, 8.45]) compared to normal OFC growth cohort.

**Conclusion:**

Infants with OFC GF are at risk for neurodevelopmental and behavioral impairment.

**Clinical trial registration:**

This study is a secondary analysis of pre-existing data from the PENUT Trial Registration: NCT01378273.

## Introduction

Providing adequate nutrition to the premature infant for the first two years is essential for neurodevelopment, the gut microbiome, bone health, and metabolism [1–4]. The most common markers of adequate nutritional status are serial measurements of anthropometrics (weight, length, and occipitofrontal circumference (OFC)). However, the definition of inadequate growth, or “growth failure,” (GF) during neonatal intensive care unit (NICU) admission is varied [5]. Furthermore, there is limited information on what constitutes optimal growth after NICU discharge.

Extremely preterm infants are at substantial risk for both GF and neurodevelopmental impairment. Early studies examining GF in extremely low birth weight (ELBW) infants defined GF based on changes in growth velocity and small for gestational age status at 36 weeks postmenstrual age (PMA) [6–8]. Greater weight gain was associated with improved Bayley Scales of Infant Development (BSID)-II mental and psychomotor subscales at 18 months [6, 8] and at 22 months [6]. Similar findings were found for OFC growth [6, 8]. However, increased weight gain or “catch-up growth” in infancy has also been associated with lower lean body mass, increased total body fat in adolescence, and increased risk for metabolic diseases later in life [9, 10]. Studies utilizing growth velocity and small for gestational age status at 36 weeks are difficult to interpret because they are not standardized and did not account for the infant’s initial growth percentiles [11].

To address this concern, many groups now consider changes in weight, length, or OFC z-score as a better measure for adequate growth [2, 12–17]. Studies of the relationship between changes in z-score during the initial hospitalization of ELBWs and neurodevelopmental outcomes show mixed results [8, 16, 17], and there has been minimal research evaluating the association of z-score changes after discharge with neurodevelopment [18].

The objective of this study was to examine changes in weight, length, and OFC z-scores from birth to discharge and from discharge to 2 years corrected age and their potential associations with neurodevelopmental and behavioral outcomes. We hypothesized that poor linear growth would be associated with adverse neurodevelopmental and behavioral outcomes at 2 years of age.

## Methods

### Patient population

This is a secondary analysis of the Preterm Erythropoietin Neuroprotection (PENUT) Trial (NCT #01378273) [19]. PENUT was a randomized, double-blinded, placebo-controlled trial of erythropoietin for neuroprotection in infants born 24 through 27 completed weeks of gestation. The study was conducted at 19 sites and 30 NICUs in the United States between December 2013 and September 2016 [19]. Infants with known chromosomal differences or congenital anomalies known to affect neurodevelopmental outcomes were excluded from enrollment. All infants enrolled in the PENUT Trial who survived and were assessed for long-term developmental outcomes were eligible for this analysis except those requiring invasive mechanical ventilation at discharge (*N* = 17), and those with hydrocephalus/ventriculomegaly on cranial ultrasound at 36 weeks postmenstrual age (PMA) (*N* = 39) as these entities are known to affect growth and OFC parameters. The PENUT Trial was approved by an institutional review board at each site. Informed parental consent was obtained before infant enrollment. This study followed the Strengthening the Reporting of Observational Studies in Epidemiology (STROBE) and Transparent Reporting of a multivariable prediction model for Individual Prognosis or Diagnosis (TRIPOD) reporting guidelines.

We collected data about maternal characteristics, pregnancy, and delivery, as well as infant characteristics including anthropometric measurements, time to regain birth weight, exposure to medications, and comorbidities during their NICU stay [19]. Severe NEC was defined as Bell’s stage 2b to 3. Severe ICH was defined as grade III or IV either unilateral or bilateral, according to Papile staging. Bronchopulmonary dysplasia (BPD) was defined as requiring nasal cannula or higher levels of respiratory support at 36 weeks PMA. Severe sepsis was defined as culture-proven bacterial or fungal sepsis resulting in blood-pressure support or substantive new respiratory support. Feeding status at discharge was defined as the method the infant was being fed (orally, gavage, or parenteral nutrition dependent). At 20 to 33 months CA, infants were evaluated by certified examiners who assessed cognitive, motor, and language development with BSID-III. All BSID-III subscales were based on the corrected age at the time of the assessment. Child Behavior Checklist (CBCL) for ages 1–5 years was administered, and T scores for emotionally reactive, anxious/depressed, somatic complaints, withdrawn, sleep problems, attention problems, aggressive behavior problems, depressive problems, anxiety problems, autism spectrum problems, attention deficit/hyperactivity problems, oppositional defiant problems, and total problem score were collected. Infants were also screened by the Modified Checklist for Autism in Toddlers (M-CHAT-R), and the total score was collected.

### Growth parameters analyzed

Weight, length, and OFC measurements were collected at birth, 14 days, discharge, and 2 years corrected age. Body mass index (BMI) was calculated at 2 years corrected age. Fenton growth curves were utilized to calculate weight, length, and OFC z-scores for growth parameters during the NICU hospitalization [20]. Weight, length, OFC, and BMI z-scores were calculated using Centers for Disease Control and Prevention growth curves at 2 years corrected age [21]. Weight, length, and OFC GF during hospitalization were defined as: z-score discharge – z-score birth ≤−0.8, as defined by the Academy of Nutrition and Dietetics [14]. Weight, length, or OFC accelerated growth during hospitalization was defined as: z-score discharge – z-score birth ≥0.8 for weight or length. Normal growth during hospitalization was defined as a z-score change of −0.799 to 0.799. Weight, length, or OFC GF from discharge to follow-up were defined as: z-score 2 years – z-score discharge ≤−0.8. Weight or length accelerated growth from discharge to follow-up was defined as: z-score 2 years – z-score discharge ≥0.8. Normal growth from discharge to follow-up was defined as a z-score change of −0.799 to 0.799. If z-score changes were >3 or <−3, they were truncated to 3 or −3 to avoid bias in likely input errors. 53 (9%) infants had one or more z-score change truncated between birth and discharge, and 80 (13.6%) infants had one or more z-score change truncated between discharge and follow-up.

### Statistical analyses

Because this study was a post-hoc analysis of a randomized control trial, it was not powered. Summary demographic variables are presented as mean and standard deviation (SD) except for days to regain birthweight which is presented as median with interquartile range (IQR). Outcome data (BSID-III, CBCL, and M-CHAT-R) were assumed to have an underlying normal (BSID-III, M-CHT) or binomial (CBCL) distribution given sample size. Z-score calculations were completed using the peditools library in R, and the cohorts were divided into GF, normal growth, and accelerated growth for weight, length, and OFC. The associations between change in z-scores from birth to discharge and BSID-III scores were visually compared using locally estimated scatter plot smoothing (loess) plot. For all inferential analyses, generalized estimating equations (GEE) with robust standard errors were used to appropriately account for potential correlation of outcomes for same-birth siblings [22]. Baseline and demographic factors were compared across growth trajectory groups using a multivariate Wald test.

GEE linear regression models adjusted for gestational age, sex, maternal education, >14 days of dexamethasone, length z-score at birth, pregnancy induced hypertension, grade III or IV intracranial hemorrhage (severe ICH), BPD, erythropoietin use, and feeding status at discharge were used to compare growth categories to neurodevelopmental and behavioral scores (BSID-III and M-CHAT-R). GEE logistic regression models were used to determine adjusted odds ratios (aOR) for having a borderline/clinical score in CBCL sub-parameters based on post-discharge growth trajectory, adjusting for gestational age, sex, maternal education, >14 days of dexamethasone, length z-score at birth, pregnancy induced hypertension, severe ICH, BPD, erythropoietin use, and feeding status at discharge. Maternal race was adjusted for in both GEE linear and logistic regression models examining growth categories from discharge to 2 years. Model outputs are presented as adjusted mean difference or aOR with 95% confidence intervals (CI). All analyses were conducted using R statistical package (Version 4.1.2, Foundation for Statistical Computing, Vienna, Austria). A *p* < 0.05 was considered statistically significant.

## Results

### Growth from birth to NICU discharge

Of the original 941 infants enrolled in the trial, 692 survived and had at least one BSID-III subscale score assessed at 2-year follow-up. After excluding infants who had mechanical ventilation at discharge (*n* = 17), ventriculomegaly (*n* = 39), and missing follow-up body measurement data (*n* = 46), *n* = 590 infants met the criteria for this secondary analysis. Maternal and child characteristics for infants with length growth cohorts are shown in Table 1, weight growth cohorts in Supplementary Table 1, and OFC growth cohorts in Table 2. Growth velocities and common co-morbidities of prematurity including necrotizing enterocolitis, severe ICH, BPD, symptomatic culture-positive sepsis, and length of stay are included. BMI z-score at 2 years did not differ between those with weight or length accelerated, normal growth, or GF during their initial hospitalization. Birth to discharge weight z-score change was positively associated with length z-score change (r^2^ = 0.16, *p* < 0.001, Supplementary Fig. 1A) and OFC z-score change (r^2^ = 0.21, *p* < 0.001, Supplementary Fig. 1C). Birth to discharge OFC z-score change was positively associated with length z-score change (r^2^ = 0.09, *p* < 0.001, Supplementary Fig. 1B).

**Table 1:** Cohort subject characteristics during the timeframe of birth to hospital discharge.

	Length GF, *n* (%)	Normal Length Growth, *n* (%)	Accelerated Length Growth, *n* (%)	*p*-value
Included infants (*n* = 590)	353	208	29	-
Maternal Characteristics				
Maternal age, years	28.6 (6.1)	29.8 (6.3)	30.7 (5.7)	0.071
Maternal education				
High School or less	123 (34.8)	53 (25.5)	7 (24.1)	0.06
Some College	98 (27.8)	74 (35.6)	10 (34.5)	
Bachelor’s or greater	83 (23.5)	63 (30.3)	9 (31.0)	
Not reported	49 (13.9)	18 (8.7)	3 (10.3)	-
Maternal race				
White	232 (65.7)	143 (68.8)	22 (75.9)	0.80
Black	84 (23.8)	44 (21.2)	4 (13.8)	
Other	24 (6.8)	16 (7.7)	2 (6.9)	
Not reported	13 (3.7)	5 (2.4)	1 (3.4)	-
Hispanic maternal ethnicity	72 (20.4)	52 (25.0)	9 (31.0)	0.30
Pregnancy induced hypertension	31 (8.8)	16 (7.7)	1 (3.4)	0.54
Infant Characteristics				
Small for gestational age	48 (13.6)	27 (13.0)	3 (10.3)	0.91
Male sex	181 (51.3)	105 (50.5)	14 (48.3)	0.92
Gestational age, weeks	25.5. (1.1)	25.8 (1.1)	26.0 (1.0)	0.99
Birth weight z-score	−0.03 (0.85)	−0.12 (0.87)	−0.16 (0.97)	0.45
Birth length z-score	−0.20 (0.94)	−0.59 (0.92)	−1.48 (1.59)	**<0.0001**
Birth OFC z-score	−0.25 (0.85)	−0.25 (0.95)	−0.13 (2.65)	0.97
Time to regain birth weight, days	9.0 (4.5)	8.6 (4.5)	9.0 (3.3)	0.69
Discharge weight z-score	−1.04 (0.82)	−0.73 (0.81)	−0.40 (0.95)	**<0.0001**
Discharge length z-score	−2.08 (1.22)	−0.91 (0.99)	0.26 (1.32)	**<0.0001**
Discharge OFC z-score	−0.91 (1.24)	−0.60 (0.91)	−0.24 (0.91)	**<0.0001**
Severe necrotizing enterocolitis	16 (4.5)	7 (3.4)	0 (0.0)	1.0
Severe intracranial hemorrhage	27 (7.6)	11 (5.3)	4 (13.8)	0.14
Bronchopulmonary dysplasia	209 (59.2)	132 (63.5)	21 (72.4)	0.090
Severe sepsis	22 (6.2)	9 (4.3)	1 (3.4)	0.64
>14 days of dexamethasone	24 (6.8)	15 (7.2)	1 (3.4)	0.64
Length of stay, days	102 (32)	101 (30)	99 (20)	0.72
Weight growth velocity, g/day	21.1 (4.3)	22.8 (4.5)	25.9 (4.0)	**<0.0001**
Length growth velocity, cm/day	0.10 (0.03)	0.20 (0.02)	0.21 (0.04)	**<0.0001**
OFC growth velocity, cm/day	0.10 (0.02)	0.11 (0.02)	0.11 (0.04)	**0.022**

GF and accelerated growth are defined as having length GF or accelerated length growth at discharge. Categorical variables are represented by *n* (%). Continuous variables are represented with mean (SD) except for time to regain birth weight which is median (IQR). *P*-values were calculated using a multivariate Wald test adjusting for treatment group except for maternal education and race, which were compared using a Chi-square test. *P*-values <0.05 are highlighted in bold.

**Table 2:** Cohort subject characteristics during the timeframe of birth to hospital discharge.

	OFC GF, *n* (%)	Normal OFC Growth, *n* (%)	Accelerated OFC Growth, *n* (%)	*p*-value
Included Infants (*n* = 590)	208	335	47	-
Maternal Characteristics				
Maternal age, years	28.2 (6.4)	29.6 (6.1)	30.8 (5.3)	0.051
Maternal education				
High School or less	62 (29.8)	108 (32.2)	13 (27.7)	0.80
Some College	64 (30.8)	99 (29.6)	19 (40.4)	
Bachelor’s or greater	52 (25.0)	91 (27.2)	12 (25.5)	
Not reported	30 (14.4)	37 (11.0)	3 (6.4)	-
Maternal race				
White	146 (70.2)	222 (66.3)	29 (61.7)	0.40
Black	45 (21.6)	77 (23.0)	10 (21.3)	
Other	14 (6.7)	22 (6.7)	6 (12.8)	
Not reported	3 (1.4)	14 (4.2)	2 (4.3)	-
Hispanic maternal ethnicity	51 (24.5)	72 (21.5)	10 (21.3)	0.56
Pregnancy induced hypertension	17 (8.2)	25 (7.5)	6 (12.8)	0.50
Infant Characteristics				
Small for gestational age	19 (9.1)	51 (15.2)	8 (17.0)	0.075
Male sex	105 (50.5)	173 (51.6)	22 (46.8)	0.80
Gestational age, weeks	25.3 (1.1)	25.8 (1.1)	26.1 (1.0)	0.96
Birth weight z-score	0.14 (0.83)	−0.15 (0.82)	−0.36 (1.09)	**<0.0001**
Birth length z-score	−0.26 (1.02)	−0.42 (0.94)	−0.75 (1.39)	**0.037**
Birth OFC z-score	0.19 (1.26)	−0.42 (0.72)	−0.90 (1.17)	**<0.0001**
Time to regain birth weight, days	9.6 (4.3)	8.4 (4.4)	8.2 (4.5)	**0.005**
Discharge weight z-score	−1.12 (0.74)	−0.83 (0.86)	−0.46 (0.86)	**<0.0001**
Discharge length z-score	−1.83 (1.30)	−1.38 (1.23)	−1.56 (1.97)	**0.0008**
Discharge OFC z-score	−1.43 (0.88)	−0.58 (0.76)	0.82 (2.00)	**<0.0001**
Severe necrotizing enterocolitis	15 (7.2)	7 (2.1)	1 (2.1)	**0.034**
Severe Intracranial Hemorrhage	19 (9.1)	19 (5.7)	4 (8.5)	0.47
Bronchopulmonary Dysplasia	129 (62.0)	204 (60.9)	29 (61.7)	0.64
Severe Sepsis	13 (6.3)	15 (4.5)	4 (8.5)	0.41
>14 days of dexamethasone	22 (10.6)	17 (5.1)	1 (2.1)	0.24
Length of stay, days	107 (27)	100 (34)	98 (22)	**0.034**
Weight Growth Velocity, g/day	20.2 (4.4)	22.6 (4.2)	24.9 (4.7)	**<0.0001**
Length Growth Velocity, cm/day	0.10 (0.04)	0.10 (0.03)	0.15 (0.05)	**0.0001**
OFC Growth Velocity, cm/day	0.09 (0.02)	0.10 (0.01)	0.14 (0.03)	**<0.0001**

GF and accelerated growth are defined as having occipitofrontal circumference (OFC) GF or accelerated OFC growth at discharge. Categorical variables are represented by *n* (%). Continuous variables are represented with mean (SD) except for time to regain birth weight which is median (IQR). *P*-values were calculated using a multivariate Wald test adjusting for treatment group except for maternal education and race, which were compared using a Chi-square test. *P*-values <0.05 are highlighted in bold.

**Fig. 1:**
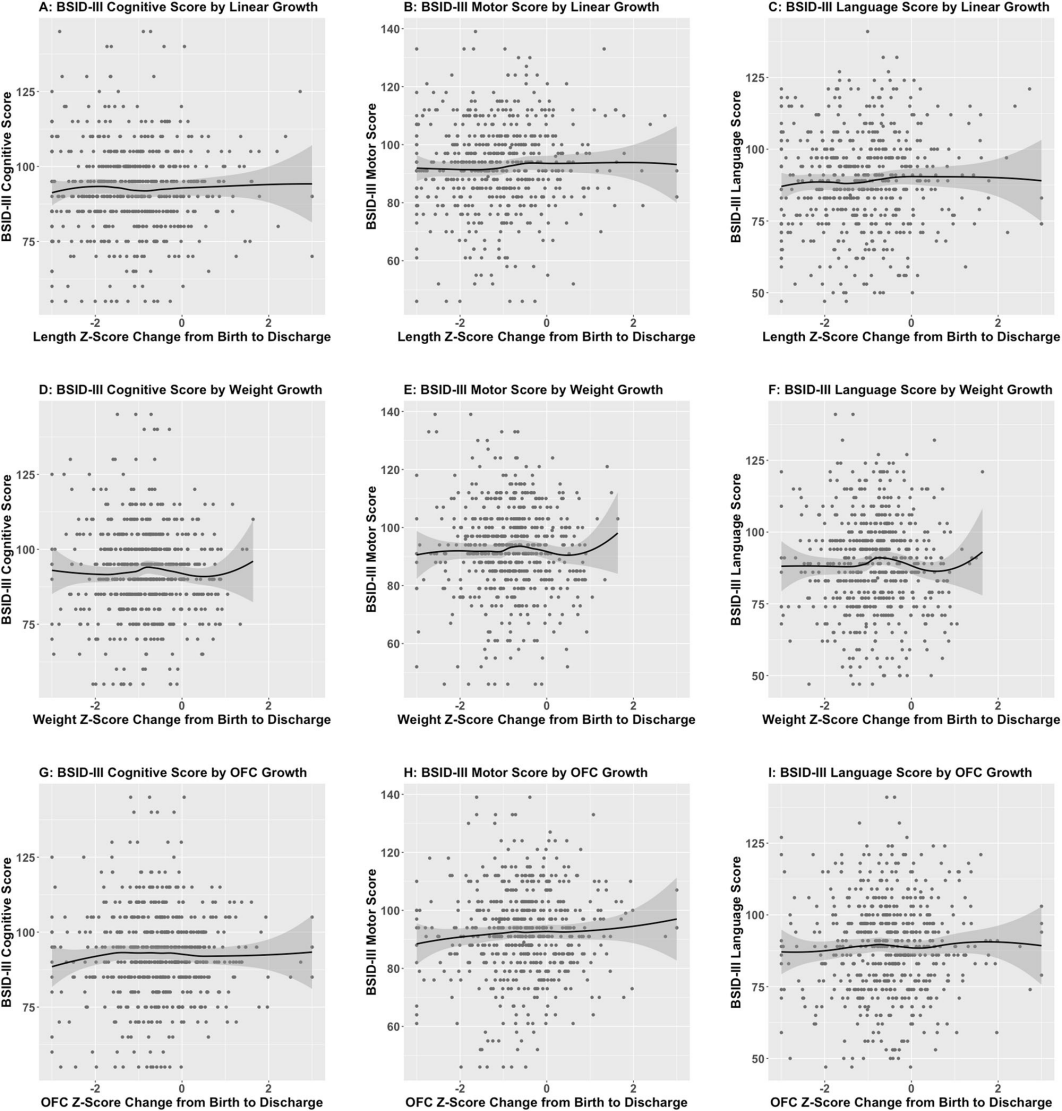
Z-score change from birth to hospital discharge for weight, length, and OFC and Bayley Scales of Infant Development (BSID-III) scores. Z-score change from birth to discharge for length (**A**–**C**), weight (**D**–**F**), and OFC (**G**–**I**) and Bayley Scales of Infant Development III (BSID-III) scores. The black line represents the local average determined using locally-estimates scatterplot smoothing (loess), and the shading represents the 95% confidence intervals.

After adjustments for comorbidities, changes in length, weight, and OFC z-score were not associated with BSID-III cognitive, motor, or language scores, either when dichotomized by GF/accelerated growth or as continuous variables (Fig. 1A–I).

**Fig. 2:**
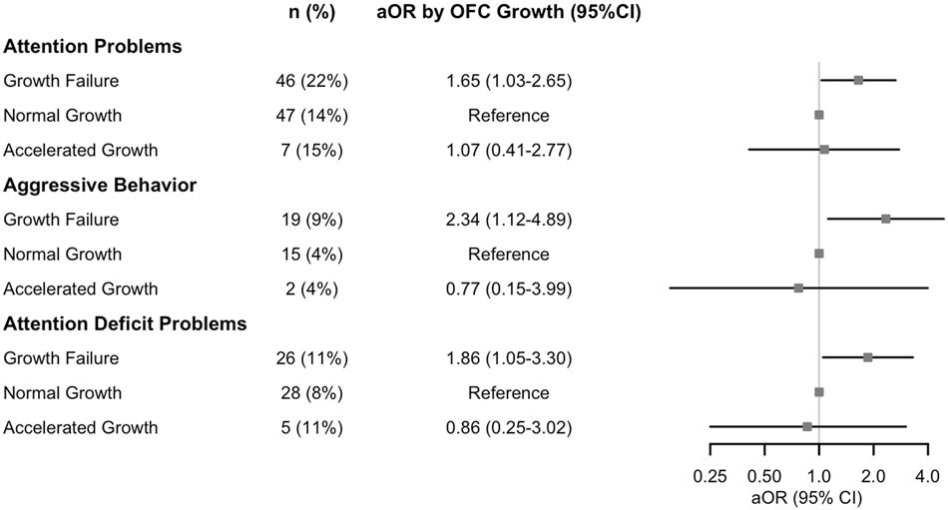
Forest plots of adjusted odds of borderline/clinical Child Behavior Checklist problem scores by in-hospital occipitofrontal circumference (OFC) growth cohorts. Adjusted odds ratio (aOR) and 95% confidence intervals (CI) for borderline/clinical score compared to those with normal growth is shown.

When examining behavioral characteristics at 2 years, infants with accelerated linear growth had a significantly lower adjusted total M-CHAT-R score compared to those with normal linear growth (−0.82 [−1.27, −0.37], *p* = 0.0004). Weight and OFC growth were not associated with total M-CHAT-R score, though there was a trend towards a lower MCHAT-R score in infants with accelerated OFC growth compared to normal OFC growth (−0.481 [−0.97, 0.01], *p* = 0.054). Compared to infants with normal OFC growth, infants who experienced OFC GF had increased adjusted odds of borderline or clinical CBCL scores for attention problems (aOR 1.65 [1.03, 2.65]; *p* = 0.038), aggressive behavior (aOR 2.34 [1.12, 4.89]; *p* = 0.024), and attention-deficit-hyperactivity symptoms (aOR 1.86 [1.05, 3.30]; *p* = 0.032) (Fig. 2). There were no significant differences in CBCL by weight or length growth cohorts.

### Growth from discharge to 2 years

Cohort characteristics are described for weight in Table 2, length in Supplementary Table 2, and OFC in Supplementary Table 3. Discharge to 2-year weight z-score change was positively associated with length z-score change (r^2^ = 0.21, *p* < 0.001, Supplementary Fig. 1D) and OFC z-score change (r^2^ = 0.24, *p* < 0.001, Supplementary Fig. 1F). Discharge to 2-year length z-score change was negatively associated with OFC z-score change (r^2^ = 0.11, *p* < 0.001, Supplementary Fig. 1E).

After adjustments for comorbidities, OFC GF at follow-up was associated with significantly lower language scores (−4.0 [−8.0, −0.1], *p* = 0.046) but not cognitive scores (−3.0 [−6.8, 0.7], *p* = 0.11) or motor scores (−3.8 [−8.4, 0.9], *p* = 0.11). There were no differences in BSID-III cognitive, motor, and language scores with changes in length or weight z-score.

**Fig. 3:**
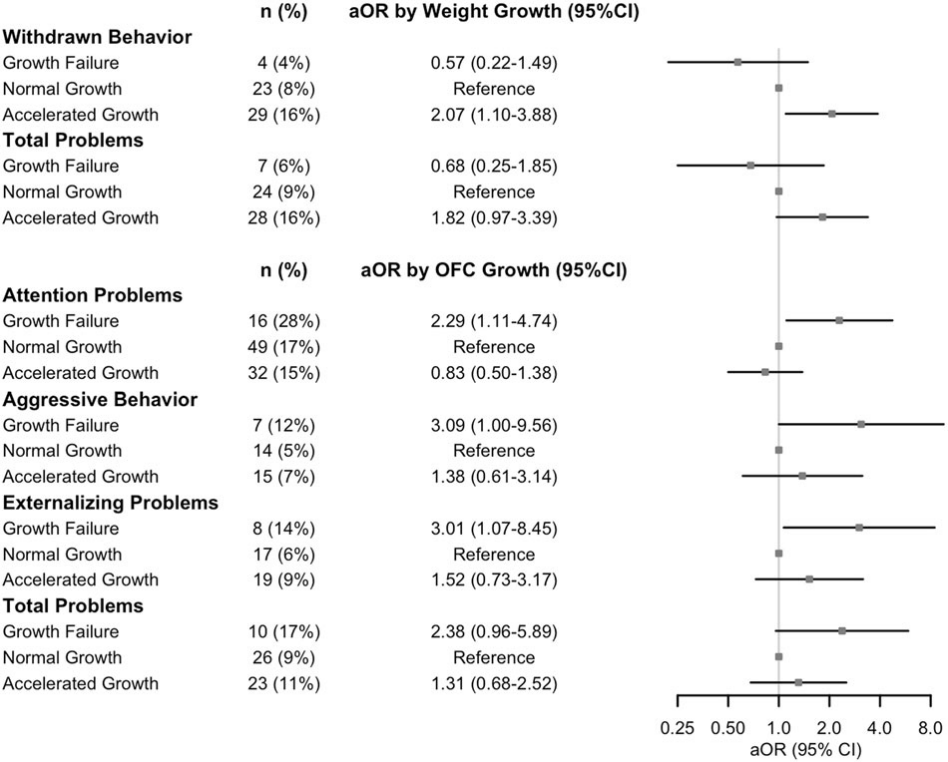
Forest plots of adjusted odds of borderline/clinical Child Behavior Checklist problem scores by discharge to 2-year follow-up occipitofrontal circumference (OFC) and weight growth cohorts. Adjusted odds ratio (aOR) and 95% confidence intervals (CI) for borderline/clinical score compared to those with normal growth is shown.

Infants with accelerated weight growth at 2 years had increased adjusted odds of borderline or clinical withdrawn behavior (aOR 2.07 [1.10, 3.88], *p* = 0.024) and a trend toward greater odds of total behavioral problems (aOR 1.82 [0.97, 3.39], *p* = 0.061) compared to those with normal weight growth (Fig. 3). Infants with OFC GF at 2 years had increased odds of attention problems (aOR 2.29 [1.11, 4.74], *p* = 0.025), aggressive behavior (aOR 3.09 [1.00, 9.56], *p* = 0.049), and externalizing problems (aOR 3.01 [1.07, 8.45], *p* = 0.037), as well as a trend towards a greater odds of total problems (aOR 2.38 [0.96, 5.89], *p* = 0.10) compared to those with normal OFC growth (Fig. 3). Behavioral outcomes on the CBCL did not differ by linear growth cohorts. There were no significant differences in M-CHAT-R scores across any growth groups between discharge and 2-year follow-up.

## Discussion

This large, multi-center post-hoc study examined the association between growth parameter z-score changes to 2-year neurodevelopmental and behavioral outcomes among extremely premature infants. Utilizing contemporary definitions of GF currently endorsed by the Academy of Nutrition and Dietetics [14], we did not find significant differences in BSID-III scores based on growth parameters obtained during hospital stay. However, OFC GF during NICU hospital stay was associated with attention problems, aggressive behavior, and ADHD symptoms. Accelerated length growth during hospitalization was associated with improved M-CHAT-R score. OFC GF from discharge to 2-year follow-up was associated with modestly decreased language score, increased attention problems, increased aggressive behavior, and increased externalizing problems. Accelerated weight z-score gain was associated with increased withdrawn behavioral problems. These findings suggest infants with OFC growth faltering during and post-hospitalization are at risk for neurodevelopmental and behavioral problems.

Studies examining growth during the NICU hospitalization and subsequent neurodevelopment are heterogeneous and conflicting. Different metrics (velocities vs. changes in z-scores vs. complex multivariate modeling of expected to observed), differing time points of evaluation (birth vs. nadir, 36 weeks PMA vs. discharge or post-discharge age), and different growth curves (Fenton vs. INTERGROWTH) have been used [2, 6–8, 12–17, 23–28]. In this study, we utilized changes in z-scores utilizing the GF definition applied by Goldberg *et al*. using the Fenton growth curve from birth to discharge [14, 20]. Brinkis et al. examining ELBW and very preterm infants utilizing these definitions did not find differences in neurodevelopmental outcomes on the BSID-II at 12 months [1]. In contrast, Rohsiswatmo et al. found a decline in weight z-score >1.2 to be associated with increased days to oral feeding [24], a known strong predictor of neurodevelopmental outcomes, and Yitayew et al. found a weight z-score change >1 to be associated with lower BSID-III scores [25]. Other studies identify poor linear growth as detrimental to cognitive and language outcomes [8, 15, 17, 26]. Yet another study found an association between a small OFC at birth and discharge with lower intelligence and motor scores in very preterm infants [27]. These results call for a standardized approach for evaluating growth in the clinical and research arenas.

This study found OFC GF from discharge to 2 years corrected age was associated with lower BSID-III language scores. There is less known about growth following NICU discharge in extremely preterm infants, but studies have found OFC to be associated with neurodevelopment. Many studies examining premature infant OFC growth following NICU discharge to various time points found an association with increased OFC growth with improved mental processing composite score intelligence quotient at 8 years [26], reading scores, lower odds of executive dysfunction, and lower odds of poor motor function at 8 years [29]. Also, increased weight velocity following discharge decreased the odds of neurodevelopmental impairment in a non-linear fashion [30]. Furthermore, a study examining growth from discharge to 2 years found gains in weight/length z-score was associated with decreased odds of cognitive impairment at 10 years old [18]. These studies suggest that growth following NICU discharge should be closely monitored by the pediatrician and the optimal growth trajectory requires further refinement.

Clinicians provide anticipatory guidance to parents on the potential developmental trajectory of their preterm infant, but the trajectory is complex and dependent on the post-discharge environment. Recent studies have questioned if the BSID-III underestimates longer-term neurodevelopmental impairment rates [31, 32]. More accurate assessments of cognitive development, executive function, psychiatric symptoms, and social function are possible as children age. A meta-analysis showed positive predictive values of evaluations performed using either the BSID or Griffiths Mental Development Scales between 1 and 3 years ranged from 20% to 89%, and negative predictive values ranged from 48% to 95% when children were assessed after 5 years of age [33]. One study examined children born at less than 32 weeks completed both a BSID-II and a BSID-III at 2 years followed by a Wechsler Preschool and Primary Scale of Intelligence Fourth Edition and found the BSID-II underestimated intelligence quotient where the BSID-III overestimated intelligence quotient [31]. A recent study published by the Neonatal Research Network assessed extremely preterm infants and healthy term reference infants’ BSID-III scores, and found using a term reference-based threshold showed higher impairment in extremely preterm infants compared to BSID-III norm-based thresholds, suggesting this may be a preferred methodology [32]. Our study found no differences in continuous BSID-III scores with in-hospital growth but found differences in OFC growth cohorts after discharge. Future studies should include follow-up to school age to accurately determine the relationships between growth and neurodevelopmental outcomes.

Although there are multiple studies showing preterm infants are at risk for behavioral and psychiatric problems [34–36], our study is of few examining behavioral outcomes and growth trajectories during and after hospitalization. In our study, poor OFC growth during NICU hospital stay and from discharge to 2 years was associated with attention problems and aggression problems. We also found there were increased withdrawn behavioral problems with accelerated weight growth after discharge. Furthermore, we identified a small (<1 point) decrease in M-CHAT-R score with accelerated linear growth compared to those with normal linear growth during hospitalization. We also noted a trend of a small decreased M-CHAT-R score with accelerated OFC growth compared to normal OFC growth. While the original M-CHAT in ELBW infants has shown poor sensitivity and positive predictive value for autism [37], the M-CHAT-R has recently been shown to be associated with neurobehavioral and CBCL outcomes, suggesting the M-CHAT-R may have utility as a developmental screen [38].

One potential reason we did not appreciate substantial differences in neurodevelopmental outcomes in infants with weight GF during NICU hospitalization is due to the methodology of examining birth weight z-score rather than weight nadir z-score. The use of weight at birth versus nadir weight is currently controversial. Weight nadir z-score may better reflect expected physiologic weight loss [39]. A study examining weight gain velocity calculations recommended the utilization of the weight nadir or day seven weight rather than birth weight [40]. Day 7 was deemed not to be significantly different than the nadir and less laborious for clinicians and researchers [40]. Our study chose to use birth weight z-score as described in Goldberg et al. as this information is standardly available in the majority of NICUs and thus is more generalizable.

We anticipated that linear growth would affect neurodevelopment and behavioral outcomes due to differences in lean body mass (bone, muscle, water). Compared to weight and OFC, length is believed to be the anthropometric measure best associated with lean body mass/fat-free mass [41]. Increased fat-free mass has been associated with improved neurodevelopmental outcomes including full-scale IQ [42], improved vocabulary, cognitive flexibility, and general cognitive function at pre-school age [43] and larger brain size at term [44]. However, studies examining the relationship between length and body composition have had small sample sizes. In contrast, in this large study of preterm infants with prospectively collected data, we did not find significant differences in BSID-III scores between in-hospital and post-discharge length cohorts. We did find a modest improvement in the M-CHAT-R score in the accelerated linear growth cohort compared to those with normal linear growth. Further large, prospective studies exploring lean mass and developmental outcomes are required.

The strength of this secondary analysis is the utilization of a large, contemporary cohort of extremely premature infants across 30 NICUs in the United States [19]. This study included a battery of developmental tests, including not only the use of BSID-III scores, but also CBCL and MCHAT-R scores [19]. This allows for a more comprehensive developmental assessment. We also applied the use of anthropometric z-score assessments which are being adopted by many quality improvement networks in the United States; thus, our results are relevant to current clinical practice. Accounting for all of these factors, we believe these results are generalizable to other US-based NICUs.

This study also has limitations. Growth was not a primary aim of the PENUT Trial, and length measurements were performed per unit protocol. Thus, we cannot be sure all length measurements were obtained on a length board which is more accurate measures of an infant’s length [45–47]. Furthermore, there was a lack of serial growth measurements during the hospital stay and post-discharge. Because of the multi-center nature of the PENUT Trial, sites followed their own nutrition protocols and we do not have information on dietary intake such as parental nutrition components, proportion of parent’s own milk, donor milk, and formula during hospital stay or after discharge.

In conclusion, extremely preterm infant growth trajectories during hospitalization showed associations with behavioral outcomes, and growth trajectories after NICU hospitalization showed associations with neurodevelopmental and behavioral outcomes. Normal OFC growth appeared to be the most neuroprotective. These findings suggest that our current nutritional definitions of weight and length GF during NICU hospitalization may not be associated with neurodevelopmental outcomes. Furthermore, nutritional studies exploring how to improve OFC growth during NICU hospitalization are imperative to optimize neonatal nutrition in preterm infants. Importantly, further research is required to determine optimal growth trajectories after NICU hospitalization, which may be most important for neurodevelopmental outcomes.

### Supplementary information

Supplementary Fig. Legend

Supplementary Tables

Supplementary Fig. 1

PENUT Trial Acknowledgments

## Data Availability

De-identified individual participant data is available through the NINDSData Archive under “Clinical Research Datasets”. The data is de-identified and a limited access data set is available through a request form on that page. Data dictionaries, in addition to study protocol, the statistical analysis plan, and the informed consent form will be included. The data will be made available upon publication of all PENUT Trial related manuscripts to researchers who provide a methodologically sound proposal for use in achieving the goals of the approved proposal.

## References

[1] Brinkis R, Albertsson-Wikland K, Tameliene R, Aldakauskiene I, Rimdeikiene I, Marmiene V, et al. Impact of early nutrient intake and first year growth on neurodevelopment of very low birth weight newborns. Nutrients. 2022;14:3682. 10.3390/nu14183682 PMC9506449 36145055

[2] Strobel KM, Del Vecchio G, Devaskar SU, Calkins KL (2023). Gut microbes and circulating cytokines in preterm infants with growth failure. J Nutr.

[3] Granger CL, Embleton ND, Palmer JM, Lamb CA, Berrington JE, Stewart CJ (2021). Maternal breastmilk, infant gut microbiome and the impact on preterm infant health. Acta Paediatr.

[4] Chinoy A, Mughal MZ, Padidela R (2019). Metabolic bone disease of prematurity: causes, recognition, prevention, treatment and long-term consequences. Arch Dis Child Fetal Neonatal Ed.

[5] Fenton TR, Cormack B, Goldberg D, Nasser R, Alshaikh B, Eliasziw M (2020). Extrauterine growth restriction” and “postnatal growth failure” are misnomers for preterm infants. J Perinatol.

[6] Ehrenkranz RA, Dusick AM, Vohr BR, Wright LL, Wrage LA, Poole WK (2006). Growth in the neonatal intensive care unit influences neurodevelopmental and growth outcomes of extremely low birth weight infants. Pediatrics.

[7] Horbar JD, Ehrenkranz RA, Badger GJ, Edwards EM, Morrow KA, Soll RF (2015). Weight growth velocity and postnatal growth failure in infants 501 to 1500 grams: 2000-2013. Pediatrics.

[8] Belfort MB, Rifas-Shiman SL, Sullivan T, Collins CT, McPhee AJ, Ryan P (2011). Infant growth before and after term: effects on neurodevelopment in preterm infants. Pediatrics.

[9] Ong KK, Loos RJ (2006). Rapid infancy weight gain and subsequent obesity: systematic reviews and hopeful suggestions. Acta Paediatr.

[10] Raaijmakers A, Jacobs L, Rayyan M, van Tienoven TP, Ortibus E, Levtchenko E (2017). Catch-up growth in the first two years of life in Extremely Low Birth Weight (ELBW) infants is associated with lower body fat in young adolescence. PLoS One.

[11] Fenton TR, Chan HT, Madhu A, Griffin IJ, Hoyos A, Ziegler EE, et al. Preterm infant growth velocity calculations: a systematic review. Pediatrics. 2017;139:e20162045. 10.1542/peds.2016-2045 28246339

[12] Rochow N, Raja P, Liu K, Fenton T, Landau-Crangle E, Gottler S (2016). Physiological adjustment to postnatal growth trajectories in healthy preterm infants. Pediatr Res.

[13] Zozaya C, Diaz C, Saenz de Pipaon M (2018). How should we define postnatal growth restriction in preterm infants?. Neonatology.

[14] Goldberg DL, Becker PJ, Brigham K, Carlson S, Fleck L, Gollins L (2018). Identifying malnutrition in preterm and neonatal populations: recommended indicators. J Acad Nutr Diet.

[15] Simon L, Theveniaut C, Flamant C, Frondas-Chauty A, Darmaun D, Roze JC (2018). In preterm infants, length growth below expected growth during hospital stay predicts poor neurodevelopment at 2 years. Neonatology.

[16] Salas AA, Bhatia A, Carlo WA (2021). Postnatal growth of preterm infants 24 to 26 weeks of gestation and cognitive outcomes at 2 years of age. Pediatr Res.

[17] Ramel SE, Demerath EW, Gray HL, Younge N, Boys C, Georgieff MK (2012). The relationship of poor linear growth velocity with neonatal illness and two-year neurodevelopment in preterm infants. Neonatology.

[18] O’Shea TM, Register HM, Yi JX, Jensen ET, Joseph RM, Kuban KCK (2023). Growth during infancy after extremely preterm birth: associations with later neurodevelopmental and health outcomes. J Pediatr.

[19] Juul SE, Comstock BA, Wadhawan R, Mayock DE, Courtney SE, Robinson T (2020). A randomized trial of erythropoietin for neuroprotection in preterm infants. N Engl J Med.

[20] Fenton TR, Kim JH (2013). A systematic review and meta-analysis to revise the Fenton growth chart for preterm infants. BMC Pediatr.

[21] Kuczmarski RJ, Ogden CL, Guo SS, Grummer-Strawn LM, Flegal KM, Mei Z (2002). 2000 CDC Growth Charts for the United States: methods and development. Vital- Health Stat.

[22] Maldonado G, Greenland S (1993). Simulation study of confounder-selection strategies. Am J Epidemiol.

[23] Cordova EG, Belfort MB (2020). Updates on assessment and monitoring of the postnatal growth of preterm infants. Neoreviews.

[24] Rohsiswatmo R, Kaban RK, Sjahrulla MAR, Hikmahrachim HG, Marsubrin PMT, Roeslani RD (2023). Defining postnatal growth failure among preterm infants in Indonesia. Front Nutr.

[25] Yitayew M, Chahin N, Rustom S, Thacker LR, Hendricks-Munoz KD. Fenton vs. Intergrowth-21st: postnatal growth assessment and prediction of neurodevelopment in preterm infants. Nutrients. 2021;13:2841. 10.3390/nu13082841 PMC8400500 34445001

[26] Belfort MB, Gillman MW, Buka SL, Casey PH, McCormick MC (2013). Preterm infant linear growth and adiposity gain: trade-offs for later weight status and intelligence quotient. J Pediatr.

[27] Selvanathan T, Guo T, Kwan E, Chau V, Brant R, Synnes AR (2022). Head circumference, total cerebral volume and neurodevelopment in preterm neonates. Arch Dis Child Fetal Neonatal Ed.

[28] Simon L, Hanf M, Frondas-Chauty A, Darmaun D, Rouger V, Gascoin G (2019). Neonatal growth velocity of preterm infants: the weight Z-score change versus Patel exponential model. PLoS One.

[29] Hickey L, Burnett A, Spittle AJ, Roberts G, Anderson P, Lee K (2021). Extreme prematurity, growth and neurodevelopment at 8 years: a cohort study. Arch Dis Child.

[30] Luo Z, You B, Zhang Y, Tang J, Zheng Z, Jia Y (2022). Nonlinear relationship between early postnatal weight gain velocity and neurodevelopmental outcomes in very-low birth weight preterm infants: A secondary analysis based on a published prospective cohort study. Front Pediatr.

[31] Flynn RS, Huber MD, DeMauro SB (2020). Predictive value of the BSID-II and the Bayley-III for early school age cognitive function in very preterm infants. Glob Pediatr Health.

[32] Green CE, Tyson JE, Heyne RJ, Hintz SR, Vohr BR, Bann CM, et al. Use of term reference infants in assessing the developmental outcome of extremely preterm infants: lessons learned in a multicenter study. J Perinatol. 2023;43:1398–405. 10.1038/s41372-023-01729-x PMC10615749 37542155

[33] Wong HS, Santhakumaran S, Cowan FM, Modi N, Medicines for Neonates Investigator Group. Developmental assessments in preterm children: a meta-analysis. Pediatrics. 2016;138:e20160251. 10.1542/peds.2016-0251 27471220

[34] Dvir Y, Frazier JA, Joseph RM, Mokrova I, Moore PS, O’Shea TM (2019). Psychiatric symptoms: prevalence, co-occurrence, and functioning among extremely low gestational age newborns at age 10 years. J Dev Behav Pediatr.

[35] Scott MN, Hunter SJ, Joseph RM, O’Shea TM, Hooper SR, Allred EN (2017). Neurocognitive correlates of attention-deficit hyperactivity disorder symptoms in children born at extremely low gestational age. J Dev Behav Pediatr.

[36] Franz AP, Bolat GU, Bolat H, Matijasevich A, Santos IS, Silveira RC, et al. Attention-deficit/hyperactivity disorder and very preterm/very low birth weight: a meta-analysis. Pediatrics. 2018;141:e20171645. 10.1542/peds.2017-1645 29255083

[37] Kim SH, Joseph RM, Frazier JA, O’Shea TM, Chawarska K, Allred EN (2016). Predictive validity of the modified checklist for autism in toddlers (M-CHAT) born very preterm. J Pediatr.

[38] Shuster CL, Sheinkopf SJ, McGowan EC, Hofheimer JA, O’Shea TM, Carter BS, et al. Neurobehavioral and medical correlates of autism screening: 2-year outcomes for infants born very preterm. J Pediatr. 2023;260:113536. 10.1016/j.jpeds.2023.113536 PMC10526642 37271496

[39] Beunders VAA, Roelants JA, Hulst JM, Rizopoulos D, Hokken-Koelega ACS, Neelis EG (2021). Early weight gain trajectories and body composition in infancy in infants born very preterm. Pediatr Obes.

[40] Fenton TR, Griffin IJ, Hoyos A, Groh-Wargo S, Anderson D, Ehrenkranz RA (2019). Accuracy of preterm infant weight gain velocity calculations vary depending on method used and infant age at time of measurement. Pediatr Res.

[41] Goswami I, Rochow N, Fusch G, Liu K, Marrin ML, Heckmann M, et al. Length normalized indices for fat mass and fat-free mass in preterm and term infants during the first six months of life. Nutrients. 2016;8:417. 10.3390/nu8070417 PMC4963893 27399768

[42] Scheurer JM, Zhang L, Plummer EA, Hultgren SA, Demerath EW, Ramel SE (2018). Body composition changes from infancy to 4 years and associations with early childhood cognition in preterm and full-term children. Neonatology.

[43] Plummer EA, Wang Q, Larson-Nath CM, Scheurer JM, Ramel SE (2019). Body composition and cognition in preschool-age children with congenital gastrointestinal anomalies. Early Hum Dev.

[44] Binder C, Buchmayer J, Thajer A, Giordano V, Schmidbauer V, Harreiter K, et al. Association between fat-free mass and brain size in extremely preterm infants. Nutrients. 2021;13:4205. 10.3390/nu13124205 PMC8708955 34959757

[45] Wood AJ, Raynes-Greenow CH, Carberry AE, Jeffery HE (2013). Neonatal length inaccuracies in clinical practice and related percentile discrepancies detected by a simple length-board. J Paediatr Child Health.

[46] Pavageau L, Rosenfeld CR, Heyne R, Brown LS, Whitham J, Lair C (2018). Valid serial length measurements in preterm infants permit characterization of growth patterns. J Perinatol.

[47] Lawn CJ, Chavasse RJ, Booth KA, Angeles M, Weir FJ (2004). The neorule: a new instrument to measure linear growth in preterm infants. Arch Dis Child Fetal Neonatal Ed.

