# Supplementary material for: Contemporary definitions of infant growth failure and neurodevelopmental and behavioral outcomes in extremely premature infants at two years of age
Source: J Perinatol. 2024 Jan 9;44(6):811–8. doi: 10.1038/s41372-023-01852-9 (PMC11161409; doi:10.1038/s41372-023-01852-9)
Supplement: Supplementary file 1 — Supplementary Fig. Legend [file 41372_2023_1852_MOESM1_ESM.docx]

**Supplementary Figure 1A-F.**  **Relationship between weight, length, and OFC z-score changes in-hospital and post-discharge utilizing Pearson correlation.** **A-C** compare weight vs. length z-score change (**A**), OFC vs. length z-score change (**B**), and OFC versus weight z-score change in-hospital (**C**). **D-F** compare discharge to two-year follow-up weight z-score change vs. length z-score change (**D**), OFC vs. length z-score change (**E**), and OFC vs. weight z-score change (**F**). The black line is from a univariate linear regression, and the shading represents the 95% confidence intervals.
