# Supplementary material for: Contemporary definitions of infant growth failure and neurodevelopmental and behavioral outcomes in extremely premature infants at two years of age
Source: J Perinatol. 2024 Jan 9;44(6):811–8. doi: 10.1038/s41372-023-01852-9 (PMC11161409; doi:10.1038/s41372-023-01852-9)
Supplement: Supplementary file 2 — Supplementary Tables [file 41372_2023_1852_MOESM2_ESM.docx]

**Supplementary Table 1.** Cohort subject characteristics during the timeframe of birth to discharge. GF and accelerated growth are defined as having weight GF or accelerated weight growth at discharge. Categorical variables are represented by n (%). Continuous variables are represented with mean (SD) except for time to regain birth weight which is median (IQR). P-values were calculated using a multivariate Wald test adjusting for treatment group, except for maternal education and race, which were compared using a Chi-square test.

|  | Weight GF, n (%) | Normal Weight Growth, n (%) | Accelerated Weight Growth, n (%) | p-value |
| --- | --- | --- | --- | --- |
| Included Infants (n=590) | 298 | 277 | 15 | - |
| **Maternal Characteristics** | | | | |
| Maternal age, years | 28.4 (6.1) | 29.8 (6.2) | 33.1 (5.1) | **0.0019** |
| Maternal education |  |  |  |  |
| High School or less | 94 (31.5) | 89 (32.1) | 0 (0.0) | 0.06 |
| Some College | 90 (30.2) | 86 (31.0) | 6 (40.0) |  |
| Bachelor's or greater | 74 (24.8) | 73 (26.4) | 8 (53.3) |  |
| Not reported | 40 (13.4) | 29 (10.5) | 1 (6.7) | - |
| Maternal race |  |  |  |  |
| White | 198 (66.4) | 185 (66.8) | 14 (93.3) | 0.40 |
| Black | 70 (23.5) | 62 (22.4) | 0 (0.0) |  |
| Other | 19 (6.4) | 22 (7.9) | 1 (6.7) |  |
| Not reported | 11 (3.7) | 8 (2.9) | 0 (0.0) | - |
| Hispanic maternal ethnicity | 77 (25.8) | 54 (19.5) | 2 (13.3) | 0.11 |
| Pregnancy induced hypertension | 16 (5.4) | 30 (10.8) | 2 (13.3) | 0.092 |
| **Infant Characteristics** | | | | |
| Small for gestational age | 16 (5.4) | 53 (19.1) | 9 (60.0) | 0.092 |
| Male sex | 158 (53.0) | 131 (47.3) | 11 (73.3) | 0.075 |
| Gestational age, weeks | 25.5 (1.1) | 25.8 (1.1) | 25.8 (1.2) | **0.015** |
| Birth weight z-score | 0.29 (0.64) | -0.38 (0.88) | -1.23 (0.92) | **<0.0001** |
| Birth length z-score | -0.13 (0.87) | -0.60 (1.09) | -1.46 (0.89) | **<0.0001** |
| Birth OFC z-score | -0.04 (0.81) | -0.43 (1.21) | -0.92 (0.74) | **<0.0001** |
| Time to regain birth weight, days | 10.2 (4.0) | 7.6 (4.3) | 3.3 (3.8) | **<0.0001** |
| Discharge weight z-score | -1.19 (0.69) | -0.62 (0.88) | -0.17 (0.76) | **<0.0001** |
| Discharge length z-score | -1.73 (1.30) | -1.35 (1.38) | -1.81 (0.97) | **0.0015** |
| Discharge OFC z-score | -0.92 (1.10) | -0.62 (1.17) | -0.60 (0.79) | **0.0087** |
| Severe necrotizing enterocolitis | 15 (5.0) | 7 (2.5) | 1 (6.7) | 0.40 |
| Severe intracranial hemorrhage | 29 (9.7) | 12 (4.3) | 1 (6.7) | 0.13 |
| Bronchopulmonary dysplasia | 173 (58.1) | 177 (63.9) | 12 (80.0) | **0.041** |
| Severe sepsis | 20 (6.7) | 11 (4.0) | 1 (6.7) | 0.45 |
| >14 days of dexamethasone | 26 (8.7) | 12 (4.3) | 2 (13.3) | 0.23 |
| Length of stay, days | 100 (33) | 102 (27) | 127 (46) | 0.097 |
| Weight growth velocity, g/day | 19.6 (3.5) | 24.0 (4.2) | 27.0 (4.5) | **<0.0001** |
| Length growth velocity, cm/day | 0.10 (0.03) | 0.10 (0.03) | 0.14 (0.02) | **<0.0001** |
| OFC growth velocity, cm/day | 0.10 (0.02) | 0.10 (0.02) | 0.11 (0.02) | **0.0008** |

**Supplementary Table 2.** Cohort comparisons during the timeframe of neonatal intensive care unit discharge to two-year infant follow-up clinic. GF and accelerated growth were categorized as having weight GF or accelerated weight growth from discharge to two-year follow-up. Categorical variables are represented by n (%). Continuous variables are represented with mean (SD) except for time to regain birth weight which is median (IQR). P-values were calculated using a multivariate Wald test adjusting for treatment group, except for maternal education and race, which were compared using a Chi-square test.

|  | Weight GF, n (%) | Normal Weight Growth, n (%) | Accelerated Weight Growth, n% | p-value |
| --- | --- | --- | --- | --- |
| Included Infants | 113 | 290 | 187 | - |
| Gestational age, weeks | 25.7 (1.1) | 25.6 (1.1) | 25.6 (1.1) | 0.68 |
| Birth weight z-score | -0.22 (1.06) | -0.06 (0.83) | 0.02 (0.76) | 0.080 |
| Birth length z-score | -0.60 (1.21) | -0.35 (0.97) | -0.33 (0.98) | 0.11 |
| Birth OFC z-score | -0.18 (1.39) | -0.25 (1.02) | -0.27 (0.81) | 0.82 |
| Male sex | 64 (56.6) | 151 (52.1) | 85 (45.5) | 0.17 |
| Maternal education |  |  |  |  |
| High School or less | 24 (21.2) | 81 (27.9) | 78 (41.7) | **0.003** |
| Some College | 40 (35.4) | 98 (33.8) | 44 (23.5) |  |
| Bachelor's or greater | 33 (29.2) | 75 (25.9) | 47 (25.1) |  |
| Not reported | 16 (14.2) | 36 (12.4) | 18 (9.6) | - |
| Maternal race |  |  |  |  |
| White | 88 (77.9) | 195 (67.2) | 114 (61.0) | **0.030** |
| Black | 12 (10.6) | 66 (22.8) | 54 (28.9) |  |
| Other | 10 (8.8) | 20 (6.9) | 12 (6.4) |  |
| Not reported | 3 (2.7) | 9 (3.1) | 7 (3.7) | - |
| Hispanic maternal ethnicity | 20 (17.7) | 58 (20.0) | 55 (29.4) | **0.03** |
| Small for gestational age | 24 (21.2) | 36 (12.4) | 18 (9.6) | **0.029** |
| Pregnancy induced hypertension | 10 (8.8) | 17 (5.9) | 21 (11.2) | 0.10 |
| Gestational diabetes | 10 (8.8) | 12 (4.1) | 10 (5.3) | 0.22 |
| Maternal obesity | 7 (6.2) | 33 (11.4) | 19 (10.2) | 0.27 |
| Days to regain birth weight, days | 8.0 (4.2) | 8.9 (4.5) | 9.2 (4.4) | **0.035** |
| Severe intracranial hemorrhage | 12 (10.6) | 19 (6.6) | 11 (5.9) | 0.22 |
| Bronchopulmonary dysplasia | 86 (76.1) | 176 (60.7) | 100 (53.5) | **0.0004** |

**Supplementary Table 3.** Cohort comparisons during the timeframe of neonatal intensive care unit discharge to two-year infant follow-up clinic. GF and accelerated growth were categorized as having length GF or accelerated length growth from discharge to two-year follow-up. Categorical variables are represented by n (%). Continuous variables are represented with mean (SD) except for time to regain birth weight which is median (IQR). P-values were calculated using a multivariate Wald test adjusting for treatment group, except for maternal education and race, which were compared using a Chi-square test.

|  | Length GF, n (%) | Normal Length Growth, n (%) | Accelerated Length Growth, n (%) | p-value |
| --- | --- | --- | --- | --- |
| Included infants | 44 | 202 | 338 | - |
| Gestational age, weeks | 25.8 (0.9) | 25.8 (1.1) | 25.5 (1.1) | 0.84 |
| Birth weight z-score | 0.21 (0.92) | 0.10 (0.76) | -0.20 (0.88) | **<0.0001** |
| Birth length z-score | -0.26 (1.10) | -0.19 (0.91) | -0.52 (1.05) | **0.001** |
| Birth OFC z-score | 0.13 (1.74) | -0.12 (0.92) | -0.36 (0.97) | **0.008** |
| Male Sex | 28 (63.6) | 111 (55.0) | 159 (47.0) | 0.053 |
| Maternal education |  |  |  |  |
| High school or less | 5 (11.4) | 59 (29.2) | 118 (34.9) | **0.010** |
| Some college | 18 (40.9) | 69 (34.2) | 92 (27.2) |  |
| Bachelor’s or greater | 16 (36.4) | 52 (25.7) | 86 (25.4) |  |
| Not reported | 5 (11.4) | 22 (10.9) | 42 (12.4) | - |
| Maternal race |  |  |  |  |
| White | 29 (65.9) | 149 (73.8) | 216 (63.9) | 0.30 |
| Black | 10 (22.7) | 34 (16.8) | 87 (25.7) |  |
| Other | 4 (9.1) | 14 (6.9) | 23 (6.8) |  |
| Not reported | 1 (2.3) | 5 (2.5) | 12 (3.6) |  |
| Hispanic maternal ethnicity | 10 (22.7) | 48 (23.8) | 72 (21.3) | 0.88 |
| Small for gestational age | 3 (6.8) | 19 (9.4) | 55 (16.3) | **0.031** |
| Pregnancy induced hypertension | 5 (11.4) | 11 (5.4) | 32 (9.5) | 0.15 |
| Gestational diabetes | 5 (11.4) | 10 (5.0) | 17 (5.0) | 0.31 |
| Maternal obesity | 5 (11.4) | 12 (5.9) | 42 (12.4) | 0.066 |
| Days to regain birth weight | 8.9 (4.6) | 8.8 (4.2) | 8.9 (4.5) | 0.94 |
| Severe intracranial hemorrhage | 8 (18.2) | 10 (5.0) | 23 (6.8) | **0.004** |
| Bronchopulmonary dysplasia | 30 (68.2) | 121 (59.9) | 205 (60.7) | 0.45 |

**Supplementary Table 4.** Cohort comparisons during the timeframe of neonatal intensive care unit discharge to two-year infant follow-up clinic. GF and accelerated growth were categorized as having weight GF or accelerated weight growth from discharge to two-year follow-up. Categorical variables are represented by n (%). Continuous variables are represented with mean (SD) except for time to regain birth weight which is median (IQR). P-values were calculated using a multivariate Wald test adjusting for treatment group, except for maternal education and race, which were compared using a Chi-square test.

|  | OFC GF, n (%) | Normal OFC Growth, n (%) | Accelerated OFC Growth, n (%) | p-value |
| --- | --- | --- | --- | --- |
| Included Infants | 58 | 295 | 215 | **-** |
| Gestational Age, weeks | 25.7 (1.0) | 25.6 (1.1) | 25.6 (1.1) | 0.93 |
| Birth weight z-score | -0.07 (1.07) | -0.12 (0.88) | 0.04 (0.77) | **0.11** |
| Birth length z-score | -0.43 (1.06) | -0.43 (1.01) | -0.33 (1.04) | 0.61 |
| Birth OFC z-score | -0.01 (1.58) | -0.32 (0.99) | -0.18 (0.95) | 0.16 |
| Male sex | 44 (75.8) | 160 (54.2) | 84 (39.1) | **<0.0001** |
| Maternal Education |  |  |  |  |
| High school or less | 18 (31.0) | 84 (28.5) | 76 (35.3) | 0.20 |
| Some college | 23 (39.7) | 95 (32.2) | 57 (26.5) |  |
| Bachelor’s or greater | 10 (17.2) | 78 (26.5) | 58 (27.0) |  |
| Not reported | 7 (12.1) | 38 (12.9) | 24 (11.2) | **-** |
| Maternal race |  |  |  |  |
| White | 36 (62.1) | 198 (67.1) | 153 (71.2) | 0.40 |
| Black | 13 (22.4) | 62 (21.0) | 48 (22.3) |  |
| Other | 6 (10.3) | 25 (8.5) | 9 (4.2) |  |
| Not Reported | 3 (5.2) | 10 (3.4) | 5 (2.3) |  |
| Hispanic maternal ethnicity | 12 (20.7) | 59 (20.0) | 57 (26.5) | 0.21 |
| Small for gestational age | 8 (13.8) | 48 (16.3) | 19 (8.8) | 0.074 |
| Pregnancy induced hypertension | 0 (0.0 | 27 (9.2) | 16 (7.4) | 1.0 |
| Gestational diabetes | 4 (6.9) | 16 (5.4) | 10 (4.7) | 0.84 |
| Maternal obesity | 4 (6.9) | 34 (11.5) | 17 (7.9) | 0.29 |
| Time to regain birth weight, days | 8.4 (4.6) | 8.5 (4.5) | 9.5 (4.3) | **0.028** |
| Severe intracranial hemorrhage | 6 (10.3) | 20 (6.8) | 14 (6.5) | **0.55** |
| Bronchopulmonary dysplasia | 40 (69.0) | 184 (62.4) | 126 (58.6) | 0.26 |
