# Supplementary figures and images for: Contemporary definitions of infant growth failure and neurodevelopmental and behavioral outcomes in extremely premature infants at two years of age
Source: J Perinatol. 2024 Jan 9;44(6):811–8. doi: 10.1038/s41372-023-01852-9 (PMC11161409; doi:10.1038/s41372-023-01852-9)

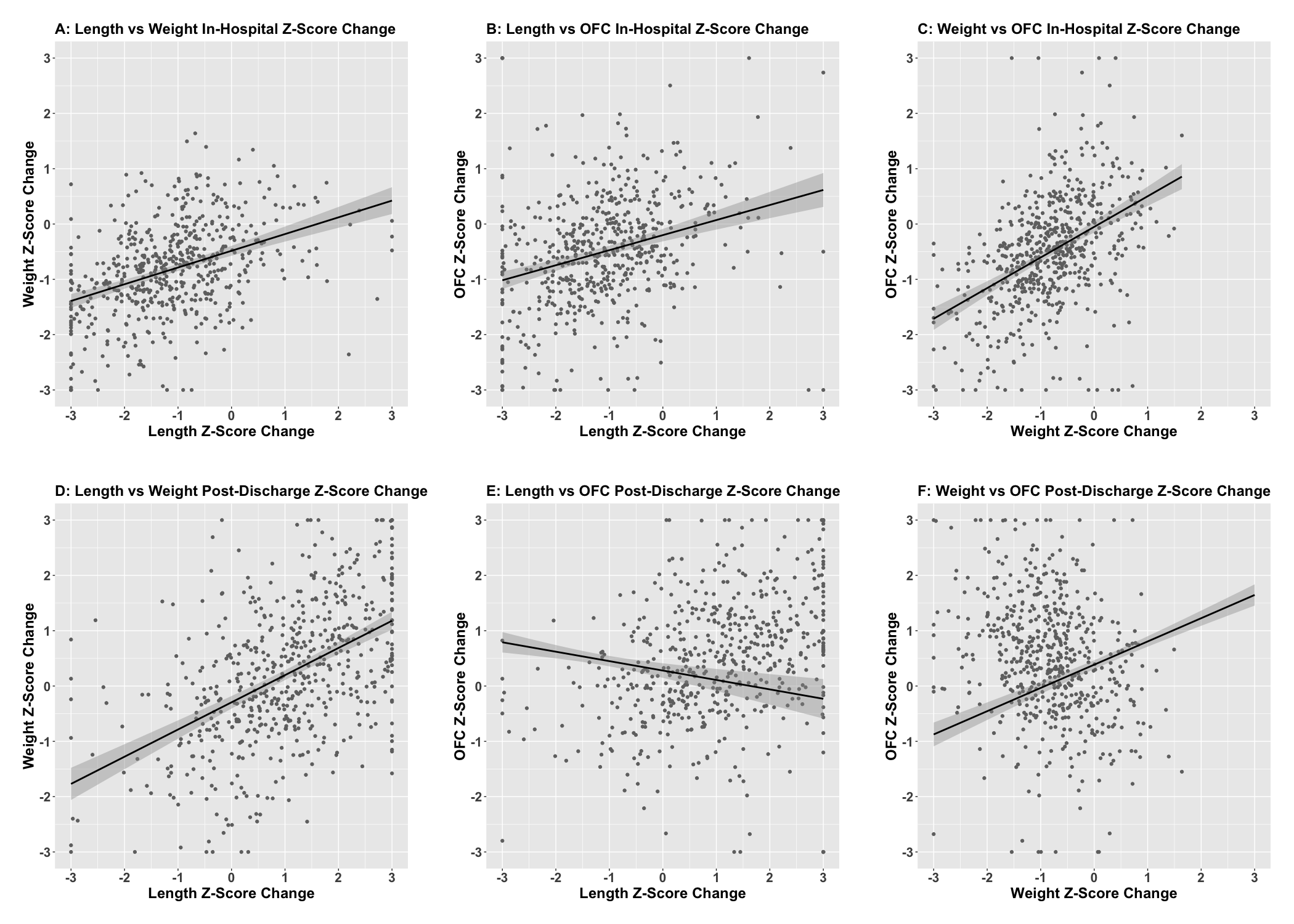

Supplement: Supplementary file 3 — Supplementary Fig. 1 [file 41372_2023_1852_MOESM3_ESM.tif]
