# Supplementary material for: Contemporary definitions of infant growth failure and neurodevelopmental and behavioral outcomes in extremely premature infants at two years of age
Source: J Perinatol. 2024 Jan 9;44(6):811–8. doi: 10.1038/s41372-023-01852-9 (PMC11161409; doi:10.1038/s41372-023-01852-9)
Supplement: Supplementary file 4 — PENUT Trial Acknowledgments [file 41372_2023_1852_MOESM4_ESM.docx]

**PENUT Acknowledgements:**

**PENUT PIs**

Dennis E. Mayock^1^, Rajan Wadhawan, MD^2^; Sherry E. Courtney, MD^3^; Tonya Robinson, MD^4^; Kaashif A. Ahmad, MBBS,MSc^5^; Ellen Bendel-Stenzel, MD^6^; Mariana Baserga, MD^7^; Edmund F. LaGamma, MD^8^; L. Corbin Downey, MD^9^; Raghavendra Rao, MD^10^; Nancy Fahim, MD^10^; Andrea Lampland, MD^11^; Ivan D. Frantz, III, MD^12^; Janine Khan, MD^13^; Michael Weiss, MD^14^; Maureen M. Gilmore, MD^15^; Robin K. Ohls, MD^16^; Jean Lowe PhD^16^, Nishant Srinivasan, MD^17^; Jorge E. Perez, MD^18^; Victor McKay, MD^19^

**PENUT Co-Investigators**

Billy Thomas, MS, MPH^3^; Nahed Elhassan, MD, MPH^3^; Sarah Mulkey, MD, PhD^3^; Vivek K. Vijayamadhavan, MD^5^; Neil Mulrooney, MD^6^; Bradley Yoder, MD^7^; Jordan S. Kase, MD^8^; Jennifer Check, MD, MS^9^; Semsa Gogcu, MD, MPH^9^; Erin Osterholm, MD^10^; Thomas George,MD^10^; Michael Georgieff, MD^10^; Camilia R. Martin, MS, MS^12^; Deirdre O’Reilly, MD, MPH^12^; Raye-Ann deRegnier^13^, Nicolas Porta, MD^13^; Catalina Bazacliu, MD^14^; Frances Northington, MD^15^; Raul Chavez Valdez, MD^15^; Patel Saurabhkumar, MD, MPH^17^; Magaly Diaz-Barbosa, MD^18^

**PENUT Research Coordinators**

John B. Feltner^1^, Isabella Esposito^1^, Stephanie Hauge^1^, Samantha Nikirk^1^, Amy Silvia^1^, Bailey Clopp^1^, Debbie Ott^2^, Ariana Franco Mora^2^, Pamela Hedrick^2^, Vicki Flynn^2^, Andrea Wyatt^3^, Emilie Loy^3^, Natalie Sikes^3^, Melanie Mason^3^, Jana McConnell^3^, Tiffany Brown^3^, Henry Harrison^3^, Denise Pearson^3^, Tammy Drake^3^, Jocelyn Wright^3^, Debra Walden^3^, Annette Guy^3^, Jennifer Nason^4^, Morgan Talbot^4^, Kristen Lee^4^, Sarah Penny^4^, Terri Boles^4^, Melanie Drummond^5^, Katy Kohlleppel^5^, Charmaine Kathen^5^, Brian Kaletka^6, 11^, Shania Gonzales^6, 11^, Cathy Worwa^6, 11^, Molly Fisher^, 11^, Tyler Richter^6, 11^, Alexander Ginder^6, 11^, Brixen Reich^7^, Carrie Rau^7^, Manndi Loertscher^7^, Laura Cole^7^, Kandace McGrath^7^, Kimberlee Weaver Lewis^7^, Jill Burnett^7^, Susan Schaefer^7^, Karie Bird^7^, Clare Giblin^8^, Rita Daly^8^, Kristi Lanier^9^, Kelly Warden^9^, Jenna Wassenaar^10^, Jensina Ericksen^10^, Bridget Davern^10^, Mary Pat Osborne^10^, Neha Talele^12^, Evelyn Obregon^12^, Tiglath Ziyeh^12^, Molly Clarke^12^, Rachel E Wegner^12^, Palak Patel^12^, Molly Schau^13^, Annamarie Russow^13^, Kelly Curry^14^, Lisa Barnhart^14^, Charlamaine Parkinson^15^, Sandra Beauman^16^, Mary Hanson^16^, Elizabeth Kuan^16^, Conra Backstrom Lacy^16^, Edshelee M. Galvis^18^, Susana Bombino^18^, Arturo E. Serize^18^, Jorge Jordan^18^, Denise Martinez^19^, Suzi Bell^19^, Corrie Long^19^

**University of Washington Data Coordinating Center**

Patrick Heagerty PhD^1^, Bryan Comstock MS^1^, Christopher Nefcy^1^, Mark A. Konodi MS^1^, Phuong T. Vu PhD^1^

**PENUT Executive Committee**

Adam Hartman MD^22^, T. Michael O’Shea MD^20^, Roberta Ballard MD^21^

**Follow Up Committee**

Mike O’Shea MD^20^, Karl Kuban MD^24^, Jean Lowe PhD^16^

**Independent Medical Monitor**

John Widness MD^23^

**Funding: NINDS**

U01NS077955 and U01NS077953

**Sites**

1. University of Washington, Seattle, Washington
2. Advent Health for Children, Orlando, Florida
3. University of Arkansas for Medical Sciences, Little Rock, Arkansas
4. University of Louisville, Louisville, Kentucky
5. Methodist Children’s Hospital, San Antonio, Texas
6. Children’s Minnesota (Minneapolis, MN)
7. University of Utah, Salt Lake City, Utah
8. Maria Fareri Children’s Hospital at Westchester, Valhalla, New York
9. Wake Forest School of Medicine, Winston-Salem, North Carolina
10. University of Minnesota Masonic Children’s Hospital, Minneapolis, Minnesota
11. Children’s Minnesota (St. Paul, MN)
12. Beth Israel Deaconess Medical Center, Boston, Massachusetts
13. Prentice Women’s Hospital, Chicago, Illinois
14. University of Florida, Gainesville, Florida
15. Johns Hopkins University, Baltimore, Maryland
16. University of New Mexico, Albuquerque, New Mexico
17. Children’s Hospital of the University of Illinois, Chicago, Illinois
18. South Miami Hospital, South Miami, Florida
19. Johns Hopkins All Children’s Hospital, St. Petersburg, Florida
20. University of North Carolina School of Medicine
21. University of California, San Francisco
22. National Institute of Neurological Disorders and Stroke
23. University of Iowa, Iowa City, Iowa
24. Boston University School of Medicine
